# Application of the hub and spokes model in improving access to cervical cancer screening in Ghana

**DOI:** 10.4314/gmj.v56i3.2

**Published:** 2022-09

**Authors:** Kofi Effah, Evans K Attivor, Bernard H Atuguba, Donatus D Adaletey, Delali A Ofori, Philip Diame, Ethel Tekpor, Comfort M Wormenor, Isaac Gedzah, Dominic Agyiri, Joseph E Amuah, Patrick K Akakpo, Jonathan M Gmanyami, Martin Adjuik, Hubert Amu, Margaret Kweku

**Affiliations:** 1 Catholic Hospital, Battor, Ghana; 2 North Tongu District Health Directorate, Ghana Health Service, Adidome, Ghana; 3 Department of Pathology, School of Medical Sciences, University of Cape Coast, Cape Coast Teaching Hospital, Cape Coast, Ghana; 4 Fred N. Binka School of Public Health, University of Health and Allied Sciences, Ho, Ghana

**Keywords:** Cervical Cancer, screening, prevention, hub and spokes model, low resource settings, North Tongu District, Ghana

## Abstract

**Objective:**

To examine the contribution of lower-level health facilities in increasing access to cervical cancer screening in the North Tongu District.

**Design:**

A descriptive cross-sectional study design was used. The Cervical Cancer Prevention and Training Centre (CCPTC) of the Catholic Hospital, Battor, served as the hub, and six health facilities (3 health centres and 3 CHPS compounds) served as the spokes. From April 2018 to September 2019, the well-resourced CCPTC trained 6 nurses at selected Community-based Health Planning and Services (CHPS) / Health Centres (HCs) (spokes) to provide cervical cancer screening services. The nurses, after training, started screening with VIA and HPV DNA testing.

**Participants:**

A total of 3,451women were screened by the trained nurses. This comprised 1,935 (56.1%) from the hub and 1,516 (43.9%) from the spokes.

**Main outcome measure:**

The detection of screen positives

**Results:**

The screen positives were 19.4% (375/1935) at the hub and 4.9% (74/1516) at the spokes.

**Conclusion:**

We have demonstrated that a hub and spokes model for cervical cancer screening is possible in limited resource settings. Designating and resourcing a ‘hub’ that supports a network of ‘spokes’ could increase women's access to cervical cancer screening. This approach could create awareness about cervical cancer screening services and how they can be accessed.

**Funding:**

None declared

## Introduction

Over 85% of the global cervical cancer burden occurs in low and middle-income countries (LMICs).^1^ In Ghana, cervical cancer is a leading cause of death among women.^2^ Every year, 2,797 women in Ghana are diagnosed with cervical cancer and 1,699 die from the disease.^3^. The estimated age-standardized incidence rate for the Greater Accra and Ashanti regions has been reported to be 24.5 and 14.0 per 100,000 respectively.^4^ This wide disparity between regions mirrors the disparity in incidence between high- and low-income countries.

This has been attributed primarily to differential access to effective screening for pre-cancer or preventative treatment, with similar disparities existing even within the same country depending on access to screening and preventative treatment.^5^ Thus, the higher rates of morbidity and mortality (27.6/100,000 vs. 7.8/100,000) due to cervical cancer in Ghana is attributable to the absence of a national coordinated cervical cancer screening and preventive treatment programme.^5^.

A large proportion of the population of Ghana lives in rural areas. These places are difficult to reach and usually do not have access to well-resourced health facilities. The higher rates of cervical cancer incidence and mortality are predicted to widen with the absence of the Human Papilloma Virus (HPV) vaccination in Ghana.^5^ Screening methods are ideal if they allow for efficient and affordable screening of all women, regardless of their economic conditions and their location.^5^ This means that the financial resources and the existing health infrastructure need to be considered carefully when choosing a cervical cancer screening modality. Although HPV testing is favoured, cheaper methods such as visual inspection with magnifying devices are more suited for low-resource settings^6^ as such services can easily be provided in deprived rural communities by trained community health personnel.

In Ghana, all screening modalities, including high-risk HPV (HrHPV) testing and preventative measures such as HPV vaccination, are available; however, the absence of a national screening programme means there is an absence of an organised framework for cervical cancer screening. Screening is thus sporadic and only available to those who access larger facilities in larger towns. Again, screening and treatment of positive lesions go hand in hand and must be available at cervical cancer screening centres. With innovative ways of bringing both Cervical Cancer Screening and Preventative Therapy (CCS&PT) to patients who need it most, there is the possibility that screening services may see a commendable increase and thus become more easily available. Still, preventative treatment may not see a commensurate increase. The complex nature of implementing a CCS&PT program in low resource settings thus needs to be anticipated, and innovative methods adopted that adapt and strengthen existing health resources.^7^

By definition, a hub-and-spoke model design arranges service delivery assets into a network consisting of an anchor establishment (hub) which offers a full array of services, complemented by secondary establishments (spokes) which offer limited service arrays, routing patients in need of more intensive services to the hub for treatment^8^. By its design, the model results in a healthcare network that consists of a main site that is better resourced, serving satellite sites that provide key basic services. The design is more efficient than those that replicate operations across multiple sites. Then again, the network of sites can be expanded when the need arises. When satellite-to-hub access becomes impractical because of geographical challenges, or numbers, additional hubs can be set up to form a multi-hub network.^8^, ^9^ Based on this model, a programme of cervical cancer screening using VIA and HPV testing was implemented in rural Malawi with good success.^10^

Our objective was to set up a self-funding and thus sustainable hub-and-spoke system of cervical cancer screening and preventative service in the North Tongu District of Ghana. The system would rely on the existing network of health centres/nurses and the Cervical Cancer Prevention and Training Centre (CCPTC) at the Catholic Hospital, Battor. Unlike the Malawian programme funded by the Scottish Government International Fund for development and involved in training health workers and setting up units to screen women, we had no support from an external partner.^10^ This study demonstrated that a ‘Hub and Spokes’ model of service delivery could be adapted to provide cervical cancer screening services in Ghana. The CHB/CCPTC served as the hub, and the already existing HC/CHPS compound served as spokes in the North Tongu District.

## Methods

### Study site

**Figure 2:**
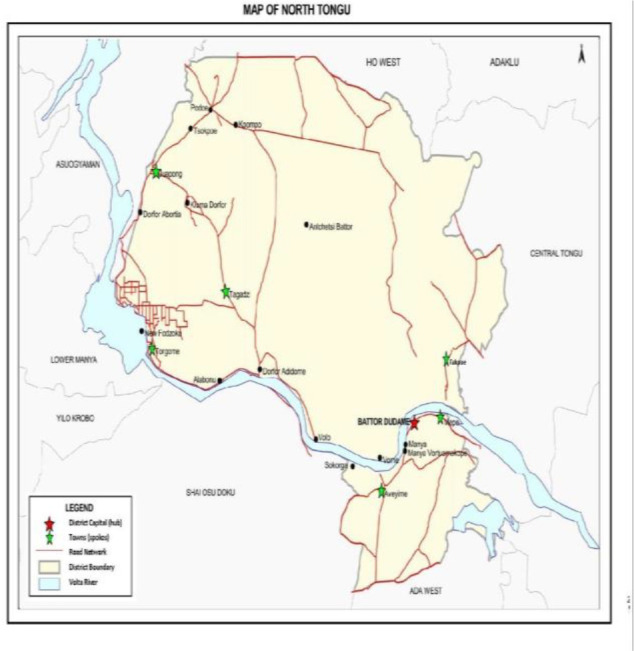
Map of North Tongu District showing the hub (red star) and the spokes (green stars)

North Tongu is one of the 18 administrative districts/municipalities in the Volta Region, with Battor as its capital. With reference to Ghana's 2010 Population and Housing Census, the population of North Tongu District (shown in Figure 2) is estimated at 89,777, representing 4.2% of the population in the Volta Region and about 0.4 per cent of Ghana's population.^10^ Females form the majority (52.7%) of the entire population. Of this population, 60 per cent live in rural areas. The district has many villages divided by the Volta River and its tributaries. The district thus has some of the most deprived and difficult to access areas in Ghana. North Tongu has one hospital (Catholic Hospital, Battor), six health centres, twenty CHPS Zones and one private clinic.^11^ Catholic Hospital, Battor, is currently the only hospital in a rural setting in Ghana that offers the full complement of services for cervical cancer prevention -vaccination, screening, and treatment for cervical pre-cancer and cancer. The Catholic Hospital, Battor, is unique because it serves a rural population and receives many clients from urban settings. With its current resources, the hospital is well-positioned as a ‘hub’ of cervical cancer screening/prevention and treatment.

The Cervical Cancer Prevention and Training Centre (CCPTC) was established in 2017. It is within the Catholic Hospital and is headed by a Gyneecologist with further training in Gynaecological Oncology. The CCPTC is a team of doctors and nurses, laboratory and records/statistics staff. The centre started training nurses, midwives and doctors in 2017.

In collaboration with the North Tongu District Health Directorate (NTDHD), the CCPTC trained selected officers for the spokes. The training was sponsored by the North Tongu District Health Directorate and Catholic Hospital, Battor. The selection of the health workers was based on their interest in running the cervical cancer prevention programme at the various health facilities.

### Study design

A descriptive cross-sectional study design was used with the CCPTC serving as the hub and six health facilities (3 health centres and three CHPS compounds) serving as the spokes. Selected interested nurses from each health facility were further trained to carry out cervical cancer screening and vaccination of eligible women immediately after training between April 2018 and September 2019.

### Sampling

The nurses were selected based on their interest to carry out cervical cancer screening at the sub-district level and the ability of their respective facilities to support them. All the officers were community health nurses and midwives stationed at the CHPS compounds/health centres of the Ghana Health Service (GHS) except for the St. Anne's Polyclinic, which is a polyclinic under the Christian Health Association of Ghana (CHAG).

All the health facilities were selected by the district health directorate based on their location in the sub-district. They were Juapong Health Centre, Tagadzi-Dorfor St. Anne's Polyclinic, Torgorme Health Centre, Fakpoe CHPS Compound, Mepe CHPS Compound, and Aveyime CHPS Compound.

### Training and data collection

All the trainees were provided with the initial resources needed for screening.

### Module 1: Basic course

Module 1 training comprised the following core competencies: Trainees learned the rudiments of cervical cancer screening, how to pass a speculum, the administrative procedures that go with a screening set up and how to perform Visual Inspection with Acetic Acid (VIA). Those who completed this module could, among other things, start cervical screening with HPV testing and VIA in their institutions. They, however, needed help with follow-up of the screen positives. At the end of the training program, each trainee outlined how they would set up screening centres in their CHPS zone/HC. They were then supported to set these centres up and were later integrated into the network of ‘spokes’ that referred cases to the ‘hub’. The six (6) facilities were then equipped to serve as primary screening sites (spokes).

### Data collection

The spokes were equipped to run a cervical cancer screening programme using Visual Inspection with Acetic Acid (VIA). Items needed, such as disposable/plastic specula, cotton swabs (which the health workers learnt to make by themselves during the training) and acetic acid, were initially provided by the Catholic Hospital, Battor and the North Tongu District Health Directorate.

The hub is fully equipped with equipment to run HPV DNA testing, perform LEEPs and radical hysterectomies.

The hub, after training, supervises the spokes, and there is good coordination between the hub and spokes. Even though the spokes are less resourced, the women in the spokes who can afford them are able to benefit from some of the services provided by the hub.

The health workers in the spokes inform the women in the communities about the services available in the hub (for example, self-sampling and HPV DNA testing and PAP smear) to the women in the communities, and they choose the services they want and can afford.

For women who want to test, samples are collected by health workers in the spokes or through self-sampling by the women themselves. The health workers in the spokes arranged for the samples (brushes) to be sent to the laboratory at the hub. The results are then sent to the spokes firstly via phone call and then later followed with printed results. The results and the management plan are then communicated to the women in the communities.

### Data analyses

Data on each client was captured on standard hospital case record forms. The data was then entered into excel and stored electronically in the CCPTC's secure databases. Data were cleaned and exported to STATA version 15.1 for analysis. Frequencies and percentages were used to describe categorical variables. Means and standard deviations were used to describe continuous variables. A 5% level of significance was used for all statistical hypothesis tests.

## Results

### Cervical cancer screening

**Figure 3:**
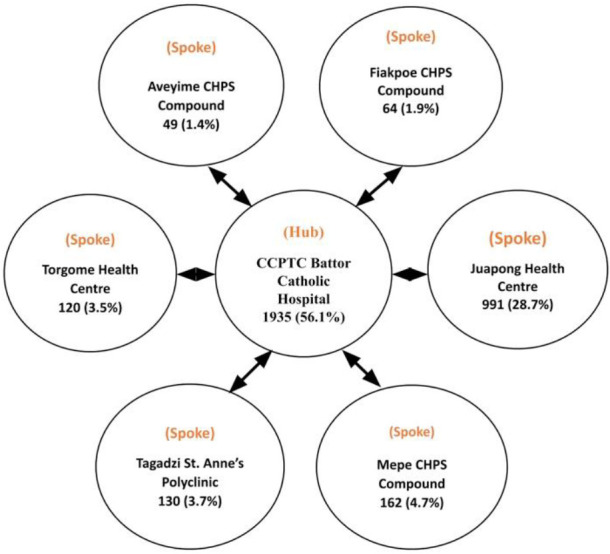
Hub and Spokes Model developed for North Tongu District

Figure 3 presents details on cervical cancer screening by the hub and spokes. The hub was the CCPTC of the Catholic Hospital, Battor. The spokes were six health facilities comprising Juapong Health Centre (HC), Torgome HC, Tagadzi St Anne's Polyclinic, Mepe CHPS Compound, Fiakpoe CHPS Compound, and Aveyime CHPS Compound.

A total of 3451 women with a mean age of 39.6±11.57 were screened. Overall, 1516 (43.9%, 95% CI: 42.3–45.6) women were screened by the spokes and 1935 (56.1%, 95% CI: 54.4–57.7) were screened by the hub (CCPTC of Catholic Hospital, Battor).

### Screening methods used

From Figure 4a, the hub used various screening methods for HPV DNA testing. These were the CareHPV, GeneXpert and Ampfire. Some clients were also screened using PAP for cytology. The various HPV DNA testing methods were combined with VIA or EVA (Colposcopy with the mobile colposcope, also known as the Enhanced Visual Assessment (EVA system).

**Figure 4a:**
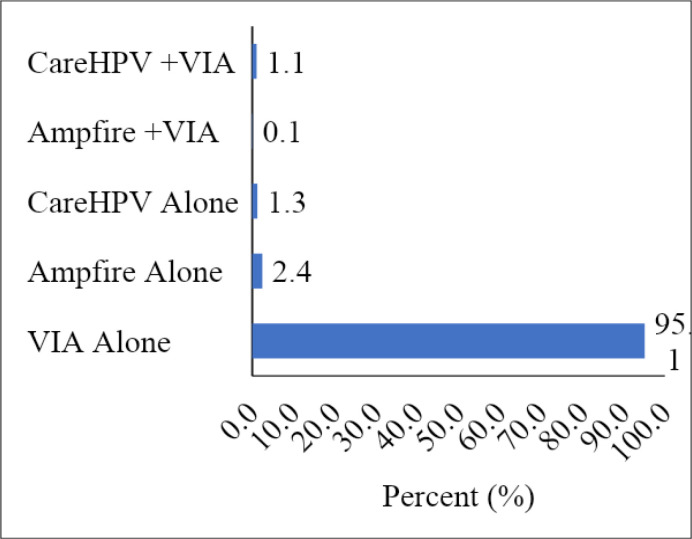
Screening method used by the Hub

The main screening methods used by the Hub either in combination or alone were CareHPV+VIA (48.3%), Ampfire+ VIA (17.8%), Care HPV + EVA (7.3%), Pap +EVA (6.4%), EVA alone (6.2%), GeneXpert +EVA (3.9%), Care HPV alone (2.2%) and Ampfire + EVA (2.0%). The rest were GeneXpert alone (1.0%), PAP +VIA (0.9%), Ampfire alone (0.7%), VIA alone (0.5%), PAP alone (0.3%), CareHPV+EVA+VIA (0.1%) and CareHPV+EVA+PAP (0.1%). For the spokes**,** the screening method used was VIA (95.1%). Samples were collected for Ampfire HPV DNA alone (2.4%), Care-HPV DNA alone (1.3%) and Ampfire HPV DNA +VIA (0.1%) and sent to the hub for testing (Figure 4b).

**Figure 4b:**
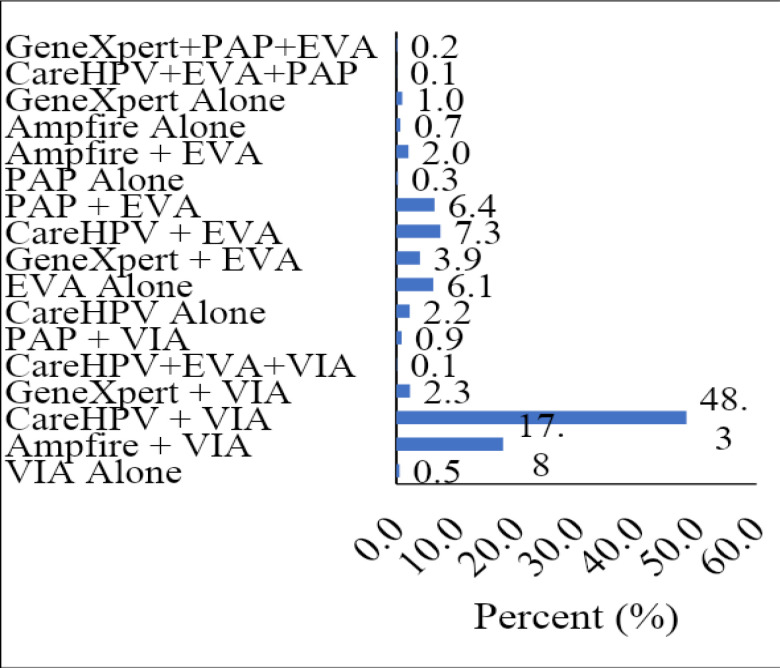
Screening method used by the spokes

Prevalence of screen positives among women screened

**Figure 5:**
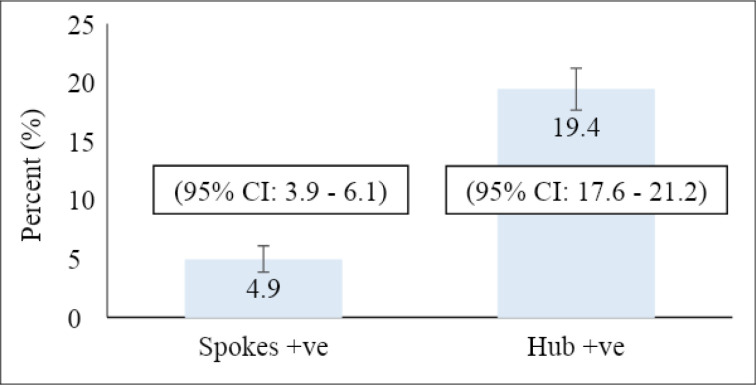
Screen positives among women screened in the hub and spokes

Out of 3451 women screened for the period, 449 (13.0%) were screen positive. Of the 1935 screened by the hub, 375 (19.4%) were screen positive. The spokes also screened 1516 women of which 74 (4.9%) were screen positives (Figure 5)

**Figure 6:**
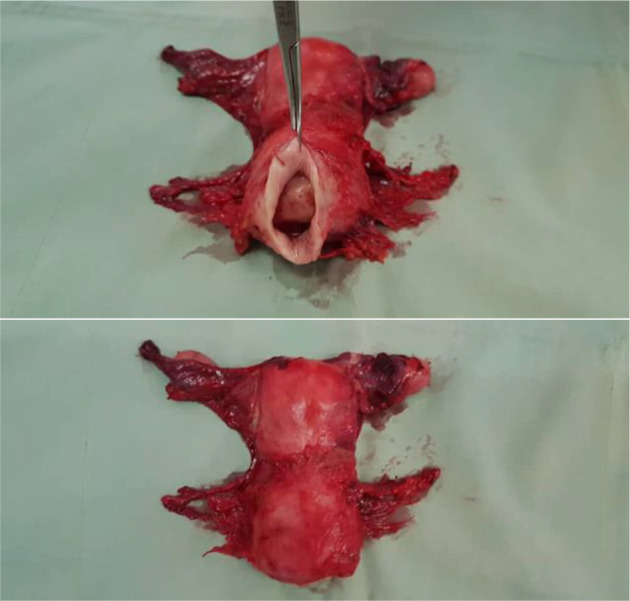
Radical hysterectomy specimen for a woman with early cervical cancer.

Figure 6 shows a radical hysterectomy specimen for a woman who was picked up by a trained nurse in one of the CHPS compounds (spoke), with early cervical cancer and the surgery was performed at the hub.

## Discussion

In this study, the North Tongu Health Directorate together with the Battor CCPTC met, discussed and selected the health facilities which should serve as spokes and also selected the nurses to be trained.

**Figure 7:**
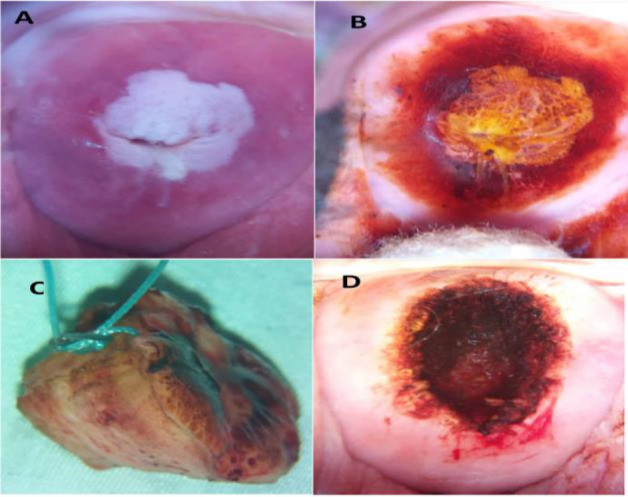
Twenty-five (25) year- old with a lesion (leukoplakia) picked up at one of the spokes. A- after application of acetic acid (spokes); B - after application of Lugol's iodine (spokes); C. LEEP specimen, stitch at 12 o'clock (hub); D. Cervix after LEEP (hub

Twenty-five (25) year-old woman with a lesion (leukoplakia) was also picked up at one of the spokes and had a LEEP done at the hub. The histopathology was CIN 1 with acanthotic squamous epithelium (Figure 7).

The selection of the health facilities was based on their location in the sub-district and being either a health centre or a CHPS compound. The selection of the nurses was based on the willingness to remain at the chosen health facility and willingness to screen after training. The number of health facilities and staff selected was based on the ability of the health directorate and CCPTC to provide resources for training and equipment and logistics needed for screening.

The CCPTC provided the nurses with basic training and successful completion of this module enabled the trainees to go and start cervical screening with HPV testing and VIA in their institutions. Trainees were taught the rudiments of cervical cancer screening, how to pass a speculum, the administrative procedures that go with a screening set up and how to perform Visual Inspection with Acetic Acid (VIA). In Malawi, a national cervical cancer, screening, prevention and treatment (CCSPT) programme was initiated in 2004 aimed at reaching a national screening rate of 80%, however, only 26.5% of their women had been screened at least once in their lifetime, and 4 out of every 10 who screened positive received appropriate treatment^12^. The VIA which is currently being used requires a time-consuming pelvic examination, which poses a challenge for large scale screening because of limited clinic space at health facilities and limited numbers of health care providers.^12^

At the end of the training programme, each trainee outlined how they were going to set up screening centres in their health facilities. They were then supported by the Health Directorate and the CCPTC to set these centres up and were integrated into the network of ‘spokes’ that referred cases to the ‘hub’. The hub and spokes screened a total of 3451 women. The hub alone screened 1935 constituting (56.1%) whilst the spokes screened 1516 (43.9%). More women could have been screened if cervical pre-cancer screening was covered by National Health Insurance Scheme in Ghana. Women had to pay from their pockets to get screened. Even though payment out of pocket was a big limitation to screening large numbers in rural areas, this made it sustainable.

The hub is well equipped with a variety of screening methods. The hub had equipment for CareHPV, Ampfire, and GeneXpert for HPV DNA testing screening, PAP for cytology and EVA Colposcopy and VIA for visual screening. The hub was, therefore, able to use a combination of screening methods such as HPV DNA testing combined with VIA, EVA colposcopy and PAP.

The spokes mainly used VIA alone (95.1%) for screening. They could also use Ampfire alone, CareHPV and Ampfire + VIA (0.1%) for some women. These samples were transported to the hub to run, indicating that the spokes can collect samples that could feed into the hub, which is better equipped.

We realised that of the 1935 women screened by the hub, 19.4% were screen positive. The spokes screened 1516 women, and 4.9% were screen positive. These results further reiterate the high prevalence of cervical cancer in Ghana, as reported by the previous studies^4^. In Malawi, Msyamboza et al., in a five-year study period, screened a total of 145,015 women, out of which 7,349 (5.1 %) and 6,289 (4.3 %) were VIA positive and suspected cancer, respectively. Overall, 13,638 (9.4 %) were detected as VIA positive or suspected cancer^12^. The hub in our study recorded higher positive cervical pre-cancer lesions because it is well equipped, and therefore, it can investigate and pick up almost all lesions. The hub used more HPV DNA testing, which has higher sensitivity than the VIA used mainly at the spokes. This is consistent with a study in India which showed that HPV DNA testing had a higher sensitivity (100%) and specificity (90.6%) compared to VIA (sensitivity = 31.6%; specificity = 87.5%)^13^. Several other studies reported similar results regarding the higher sensitivity of HPV DNA compared to VIA.^14^, ^15^, ^16^ The hub is a well-known hospital for gynaecological and cancer management; therefore, it receives many referrals from other parts of the country. It is also a referral hospital in the country for cervical cancer screening. To assess the need to adopt a national prevention strategy, the Hub and Spoke Model was identified and recommended to reach the larger rural populace who are pre-dominant victims of cervical cancer 17. Comparing the use of VIA alone, the VIA positivity rate at the spokes 4.9% is comparable to 3.33% reported in Cameroon.^18^

## Conclusion

We have demonstrated that a hub and spokes model for cervical cancer screening is possible in a limited resource setting by identifying suitable spokes and strategically positioning them to provide relevant services. In our model, the staff of the spokes were identified by the organisation in which they worked, and the selected staff were willing to remain at the selected health facility to conduct screening. Training of staff in basic VIA screening was essential for the establishment of the spokes. The spokes also needed logistic support from the hub and health administration to ensure uninterrupted routine screening. They also required laboratory support from the hub to be able to use other screening methods apart from VIA.

With a commitment by government and development partners, this model may be replicated in Ghana and other low- and middle-income countries. This will help augment efforts towards increasing access to screening, diagnosis and treatment services.

## Figures and Tables

**Figure 1:**
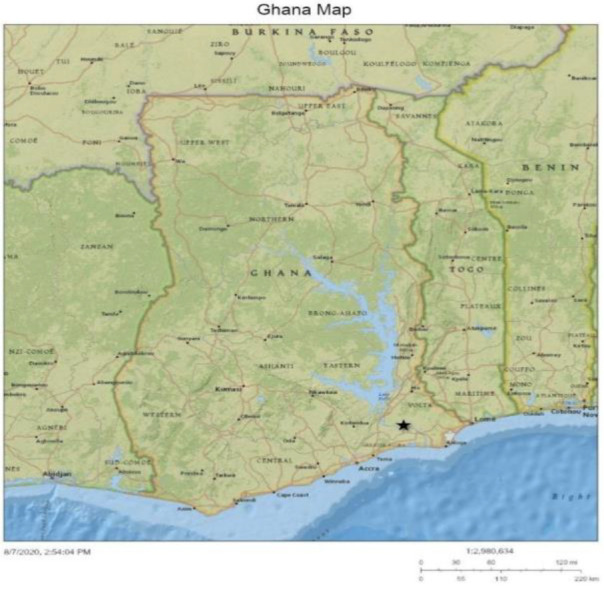
Map of Ghana showing the location of the North Tongu District (Black Star)
